# A Rapid and Sensitive Method to Measure the Functional Activity of Shiga Toxins in Human Serum

**DOI:** 10.3390/toxins7114564

**Published:** 2015-11-04

**Authors:** Valentina Arfilli, Domenica Carnicelli, Gianluigi Ardissino, Erminio Torresani, Gaia Scavia, Maurizio Brigotti

**Affiliations:** 1 Dipartimento di Medicina Specialistica, Diagnostica e Sperimentale, Sede di Patologia Generale, Università di Bologna, Via San Giacomo 14, 40126 Bologna, Italy; E-Mails: arfilli.v@libero.it (V.A.); domenica.carnicelli@unibo.it (D.C.); 2 Center for HUS Control, Prevention and Management, Fondazione IRCCS Ca’ Granda Ospedale Maggiore Policlinico, 20122 Milan, Italy; E-Mail: ardissino@centroseu.org; 3 Unit of Microbiology, Fondazione IRCCS Ca’ Granda Ospedale Maggiore Policlinico, 20122 Milan, Italy; E-Mail: e.torresani@policlinico.mi.it; 4 Istituto Superiore di Sanità, 00161 Rome, Italy; E-Mail: gaia.scavia@iss.it

**Keywords:** hemolytic uremic syndrome, Shiga toxin-producing *Escherichia coli*, eukaryotic protein synthesis, Raji cells

## Abstract

Shiga toxins (Stx) have a definite role in the development of hemolytic uremic syndrome in children with hemorrhagic colitis caused by pathogenic Stx-producing *Escherichia coli* (STEC) strains. The dramatic effects of these toxins on the microvasculature of different organs, particularly of the kidney, are well known, whereas there is no consensus on the mechanism by which Stx reach the endothelia of target organs and/or indirectly injure these body sites. We hereby describe a quick (4 h), radioactive, Raji cell-based method designed for the detection of Stx in human sera. The assay monitors the translation impairment induced by these powerful inhibitors of protein synthesis, which are identified properly by neutralizing their activity with specific monoclonal antibodies. By this method, we detected for the first time the functional activity of Stx in sera of STEC-infected patients during hemorrhagic colitis. Recent research has pointed to a dynamic process of Stx-induced renal intoxication in which concurrent and interactive steps are involved. Our rapid and specific method could be useful for studying the kinetics of Stx during the natural course of STEC infection and the interplay between Stx activity in serum and Stx presence in different blood fractions (neutrophils, monocytes, platelets, leukocyte-platelet aggregates, microvesicles, lipoproteins).

## 1. Introduction

Hemolytic uremic syndrome (HUS) is the main cause of acute renal failure in early childhood and is often the sequela of enteritis caused by Shiga toxin-producing *Escherichia coli* (STEC), hence the denomination of diarrhea-associated HUS [1,2,3]. The two main toxin types elaborated and released by STEC are Shiga toxin 1 (Stx1) and Shiga toxin 2 (Stx2) [4]; the latter is more frequently associated with HUS, as clearly demonstrated in epidemiological studies [5]. Stx are powerful inhibitors of protein synthesis in sensitive cells, since they irreversibly damage ribosomes by removing a single adenine residue from the large ribosomal RNA [6,7]. STEC infections in humans give rise to a spectrum of clinical manifestations, from watery diarrhea or bloody diarrhea to the severe and life-threatening HUS [1]. Stx and STEC have different concurring roles in the pathogenesis of STEC-related diseases: (i) bacteria are confined to the gut, and their intimate adhesion to the epithelial lining of the bowel is principally related to watery diarrhea [8,9]; (ii) toxins cross the intestinal epithelial barrier and bind to specific glycolipid receptors, namely globotriaosylceramide (Gb3Cer) and globotetraosylceramide (Gb4Cer) [10], expressed on the microvasculature of the gut, causing the development of bloody diarrhea [8,9]; (iii) Stx escaping the capture by intestinal endothelial cells reach the kidney through the blood stream and bind to Gb3Cer and Gb4Cer on glomerular endothelial cells; the latter phenomenon is considered of prime importance in the onset of HUS [4,8,9].

Although the mode of delivery of Stx from the bowel to the kidney has been extensively investigated, the exact mechanism by which Stx in blood trigger the transition from bloody diarrhea to HUS is still unknown. Stx are capable of binding to several blood components, including platelets [11,12,13], monocytes [14,15], neutrophils [16,17,18], erythrocytes [19], leukocyte- and platelet-derived microvesicles [20] and lipoproteins [21], and these interactions have variable impacts on the pathogenetic mechanisms underlying the onset of HUS. On the other hand, free Stx2 has been detected in sera of STEC-infected patients during the prodromal intestinal phase before the onset of HUS [22] and in very low amounts in sera of patients with overt HUS [23]. The detection methods used in these studies relied on very sensitive ELISA [22,24], which correctly identified the toxins without giving any information on their activity. This point is particularly important, since in human blood, a protein is present (human serum amyloid P component, HuSAP) that binds to Stx2 and impairs its toxic activity, hence protecting target cells [25,26,27]. In this respect, the detection of free Stx2 in patients’ blood represents an important finding, although it does not allow one to conclude that the toxins expressed their activity on target cells during the pathogenesis of HUS. It is worth noting that active functional Stx have never been found in patients with HUS by means of assays based on the intoxication of sensitive cells (Vero cells, human umbilical vein endothelial cells) [28,29,30]. No attempts have yet been made to investigate the toxic activity of serum free Stx in patients with bloody diarrhea before the onset of HUS. To gain information on this topic, we took advantage of the great sensitivity of Raji cells to Stx1 and Stx2 and of the very fast kinetics of intoxication [31]. The cell model appears suitable for routine daily determinations: Raji cells are easy to obtain in large amounts, and despite the fact that they were derived more than 45 years ago from a Nigerian patient with Burkitt lymphoma [32], the genome seems to have remained relatively stable after decades of continuous cultivation [33]. Here, we describe a quick and reproducible method to detect the toxic activity of Stx1 and Stx2 in human serum. The assay is quite specific, since it measures the inhibition of protein synthesis induced by Stx in cells, the hallmark of the toxic action of these powerful bacterial products.

## 2. Results and Discussion

### 2.1. Setup of Protein Synthesis Assays with Raji Cells

Many different radioactive methods have been described to measure the rate of protein synthesis in whole cells in the presence of a labeled amino acid. Since 2001, we have used a method described by Petronini and colleagues [34] and applied it to adherent cells, such as human umbilical vein endothelial cells [7,35,36,37], or cells in suspension, such as human neutrophils, Raji cells or HL-60 cells [31,38]. However, the method is time consuming and quite laborious since many steps are needed to ensure a proper separation between the labeled proteins and the unreacted free amino acid precursor ([^3^H] leucine), which might be present within the cells and in the medium. The steps required by this method are: (i) the incubation of the cell monolayer or the suspended cells with the studied compound or specimen; (ii) the addition of [^3^H] leucine, followed by a 1-h incubation at 37 °C; (iii) three washings with ice-cold phosphate buffered saline (PBS) or Earle’s salt solution to remove free [^3^H] leucine; (iv) precipitation of cellular proteins (5 min on ice) with ice-cold 10% (*v*/*v*) trichloroacetic acid (TCA); (v) two washings with ice-cold 10% (*v*/*v*) TCA, each followed by incubation for 5 min on ice; (vi) the solubilization of the denatured cellular proteins in 0.2 M KOH; and (vii) the measure of their radioactivity in a liquid-scintillation β counter. Obviously, the procedure is more tedious with cells in suspension, since every step requires a centrifugation run: 5 min at 200× *g* or 10 min at 13,000× *g* after washing with PBS or TCA, respectively. The multi-step procedure renders the assays prone to errors in the determination of the labeled amino acid incorporation into proteins, hence affecting the measure of its inhibition. For this reason, the radioactive counts must be related to the total protein content of each sample measured by the Bradford assay [39], and the results must be expressed as cpm/mg of cellular protein [34].

**Scheme 1:**
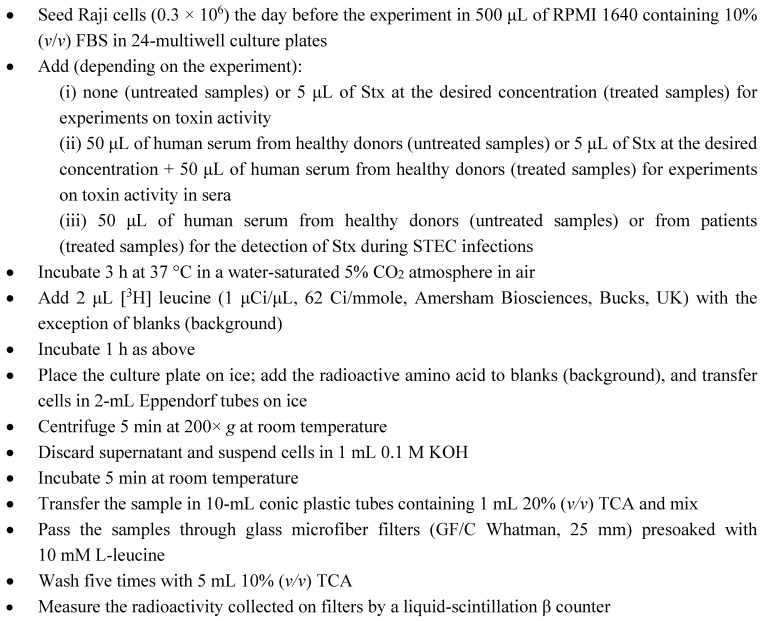
Final protocol for Stx detection through a radioactive translation assay with Raji cells.

**Figure 1:**
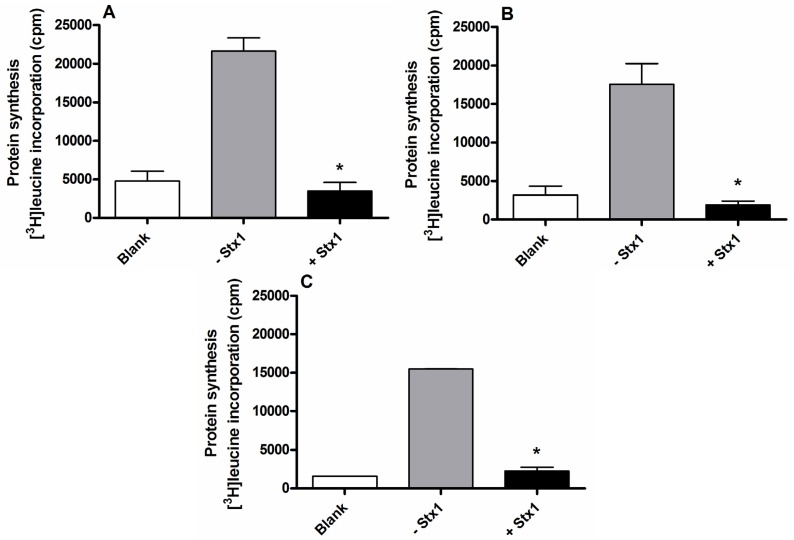
Detection of Stx through the radioactive protein synthesis assay with Raji cells. The old procedure was point-by-point described in the text and modified as follows: (**A**) no centrifugation runs, addition of a filtration step upon incubation at 90 °C of the denatured proteins; (**B**) single centrifugation run, addition of a filtration step upon incubation at 90 °C of the denatured proteins; (**C**) single centrifugation run, addition of a filtration step of the denatured proteins upon treatment of cells with KOH. Blank values were obtained by addition of the labeled amino acid on ice at the end of the incubation steps. Where indicated, 150 pM Stx1a was added. * *p* < 0.05 as compared to control cells (*t*-test).

The first objective of our study was the realization of a technically-sound procedure composed of the lowest number of steps required to achieve the abovementioned goals. We chose Raji cells, which express Gb3Cer [40], the specific receptor for Stx, and show a very quick response to these bacterial toxins, since protein synthesis was completely inhibited after a 3-h incubation with picomolar concentrations of Stx [31]. To simplify the procedure described above, the determination of protein synthesis inhibition in Raji cells challenged with Stx1a (150 pM for 3 h) was performed with the following modifications: (i) the centrifugation steps were omitted; (ii) a further incubation (10 min at 90 °C) of the TCA-precipitated proteins was introduced; and (iii) TCA-precipitated proteins were collected on glass microfiber filters (GF/C Whatman). The incubation step was required in order to cleave the bond between tRNAs and amino acids, thus reducing background values (blank). As shown in Figure 1A, protein synthesis was detectable and significantly inhibited by Stx, even though the protocol showed a main drawback, since the filtration step required several hours due to the huge amount of proteins present in the fetal bovine serum (FBS) added to the cell culture medium. To circumvent this problem, a single centrifugation run was introduced at the end of the incubation with toxins to eliminate the excess of proteins. Raji cells were resuspended in PBS and the procedure repeated as described above. The results were quite similar (Figure 1B), but in this case, the filtration time was very short (less than 2 min per sample). Finally, the definitive protocol was further simplified since cells were resuspended in 0.1 M KOH instead of in PBS. This allowed the removal of the incubation step at 90 °C, because the bonds connecting amino acids and tRNAs are broken by the alkaline pH at room temperature. Protein synthesis was efficiently measured and completely inhibited by Stx1a (Figure 1C). In conclusion, almost identical results have been obtained with the different approaches shown in Figure 1, even though the simpler and most rapid method is that in panel C (detailed description in Scheme 1).

### 2.2. Detection of Shiga Toxins in Human Serum by the Raji Cells Protein Synthesis Assay

**Figure 2:**
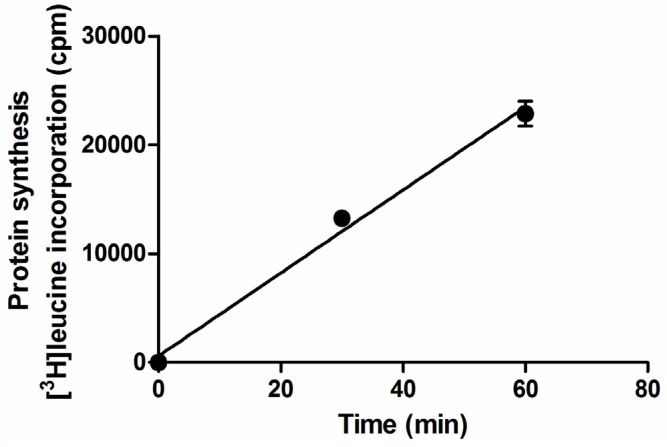
Time course of the translation in Raji cells in the presence of human serum added to complete medium (RPMI 1640 containing 10% (*v*/*v*) FBS). The experiments were performed according to Scheme 1. The SD values (*n* = 3) of single points are indicated.

The experiment described in Scheme 1 was repeated 10 times in duplicate in the presence of 50 μL human serum from healthy donors added to complete medium (RPMI 1640 containing 10% (*v*/*v*) FBS), giving a mean incorporation of 25,449 cpm. The CV(%) (coefficient of variation), calculated as SD/mean × 100, was 7.85%, and the mean percentage of background signal was 2.5%. The limit of detection was determined by the addition of the CV(%) to the mean percentage of background signal. On this basis, inhibitions of protein synthesis above 10% upon challenge of Raji cells with Stx-containing human sera were considered significant. The viability of Raji cells used in the assays (95.8% ± 1.8%; mean ± SD, *n* = 10) was measured before seeding by the trypan blue dye exclusion test. Moreover, the short incubation time (4 h) strongly reduces further effects on cell viability or proliferation after treatment with toxins rendering the assay quite specific for the determination of protein synthesis inhibition in whole cells. The results obtained in Figure 2 following the final protocol (Scheme 1) also showed that the protein synthesis rate is optimized for efficiency and linearity (*r* = −0.99) up to 60 min.

**Figure 3:**
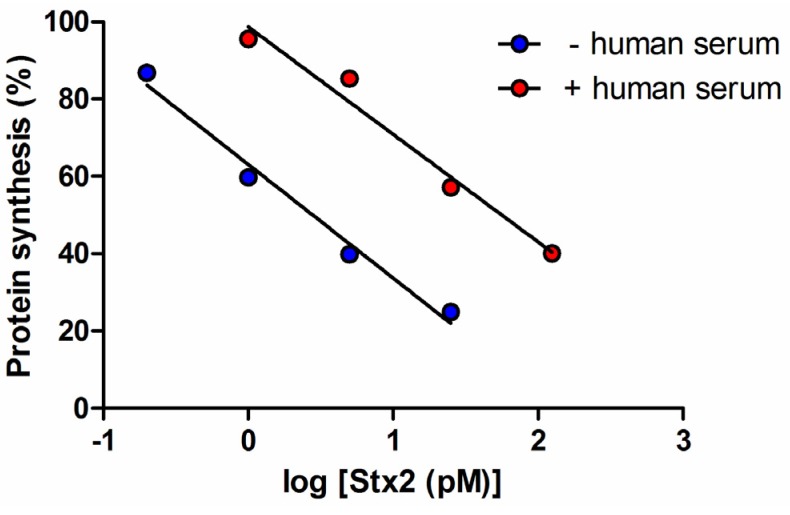
IC_50_ of Stx2a on Raji cells’ protein synthesis in the absence and in the presence of human serum from healthy donor 2 (see Table 1) added to complete medium (RPMI 1640 containing 10% (*v/v*) FBS).

The addition of different concentrations of both toxins to culture media allowed the calculation of similar IC_50_ on Raji translation for Stx1a (0.8 pM; 54.4 pg/mL; *r* = −0.97) and Stx2a (2.2 pM; 149.6 pg/mL; *r* = −0.99). The values are quite similar to those obtained by Quinones and colleagues [41] by using a very sensitive method based on a Vero cell line harboring a destabilized variant (t_1/2_ = 2 h) of the enhanced green fluorescent protein (d2EGFP) to monitor the Stx-induced inhibition of protein synthesis. Conversely, the presence of human sera from healthy donors did not significantly change the IC_50_ of Stx1a (0.3 pM; 20.4 pg/mL; *r* = −0.92), whereas, as expected, that of Stx2a showed a marked increase (IC_50_ ranging between 40 and 75 pM; 2.7–5.1 ng/mL) caused by the known Stx2 inhibitor present in human sera (HuSAP) and not in FBS [25], as depicted in a representative experiment (Figure 3) and summarized in Table 1. Although its concentration in serum is relatively stable, circulating HuSAP has been reported to be lower in females and infants, and slight variations exist among different subjects [27,42]. This would have an impact on the sensitivity of the assay, hence explaining the differences in Stx2 activity observed in Table 1. On the other hand, HuSAP is not an acute phase protein, thus differences between donors or patients in Stx2 neutralizing activities of their sera reflect the individual behaviors measured by the proposed assay. In conclusion, the limit of detection (concentration of toxins giving 10% inhibition of protein synthesis) of Stx in the presence of human sera, calculated by the reported plots (see above and Table 1), was ~2 pg/mL (~0.03 pM) for Stx1a and ~100 pg/mL for Stx2a (~1.5 pM).

**Table 1:** Effect of human serum on the detection of Stx2a by the Raji cell translation assay.

Treatment	IC_50_ (pM)	Fold-Increase	Pearson Coefficient (r)
**Stx2a**	2.2	-	−0.99
**Stx2a + human serum (donor 1)**	39.8	18.1	−0.89
**Stx2a + human serum (donor 2)**	44.2	20.1	−0.99
**Stx2a + human serum (donor 3)**	74.9	34.0	−0.99

### 2.3. Detection of Shiga Toxins in Human Serum from Patients Infected by Shiga Toxin-Producing E. coli Using the Raji Cells Protein Synthesis Assay

Although in the presence of human sera, the extent to which Stx2a inhibited protein synthesis in Raji cells was lower with respect to Stx1a, we focused on patients with bloody diarrhea with detectable Stx in feces searching for serum toxic activity due to Stx. The inhibitions of protein synthesis induced by patients’ sera were calculated by referring to controls run in the presence of human sera. The addition to controls of pooled sera from at least three healthy donors is preferred to minimize any individual contribution affecting the rate of protein synthesis.

**Figure 4:**
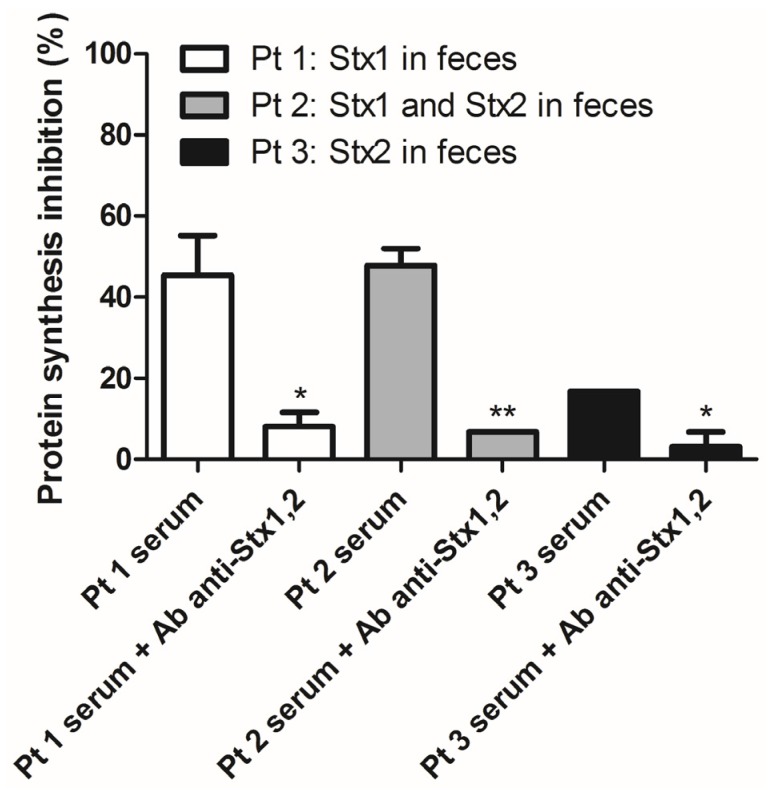
Detection by the Raji cell protein synthesis assay of Stx in sera from patients with concomitant fecal Stx during Stx-producing *Escherichia coli* (STEC)-induced bloody diarrhea. The experiment was performed in duplicate. The presence of the monoclonal antibodies (10 μg) to Stx1a and Stx2a did not affect the controls. * *p* < 0.05, ** *p* < 0.01 (*t*-test).

The results shown in Figure 4 clearly demonstrated the presence of functional Stx1 and Stx2, during the prodromal intestinal phase, detected by the assay described in the present paper. Moreover, data have been validated by the addition of mouse monoclonal IgG to Stx1 (Stx1-13C4) and Stx2 (Stx2-BB12) in order to demonstrate the causal relationship between protein synthesis inhibition and the presence of these specific bacterial toxins in patients’ blood. The simultaneous addition of the two monoclonal antibodies to the same sample was preferred in order to reduce the consumption of the valuable sera, since many other laboratory determinations need to be performed using the same specimen. The characteristics of the three representative patients are shown in Table 2.

**Table 2:** Characteristics of the patients with STEC-induced bloody diarrhea.

Patients	Age (years)	Gender	Bloody Diarrhea (days)	Detection of Shiga Toxins in Feces	
				Enrollment Assay ^a^	RT-PCR ^b^
**1**	0.8	F	4	Stx1+	n.d. ^c^
**2**	2.3	F	5	Stx1+ Stx2+	Stx1+ Stx2+ eae+
**3**	14.2	F	4	Stx2+	Stx2+ eae+

^a^ Rapid screening tests were used as the enrollment assay: an immunochromatographic test to detect Stx (Patient 1) or PCR-based reverse dot blot for the detections of *stx1* and *stx2* genes (Patients 2 and 3); ^b^ RT-PCR for the main virulence genes (*eae*, *stx1* and *stx2*) was used to confirm the results of the enrollment assay. ^c^ not determined.

## 3. Experimental Section

### 3.1. Toxins

*E. coli* C600 (H19J) and *E. coli* C600 (933W), which produce Stx1a and Stx2a, respectively, were kindly supplied by Dr. Alison O’Brien (Department of Microbiology and Immunology, Uniformed Services University of the Health Sciences, Bethesda, MD, USA). Stx1a was purified by receptor analogue affinity chromatography on globotriose-Fractogel (IsoSep AB, Lund, Sweden) [43]. Stx2a was purified on (Galα1-4Galß-O-spacer)-BSA-Sepharose 4B (Glycorex AB, Lund, Sweden) according to [44]. In both cases, a passage through ActiClean Etox columns (Sterogene Bioseparations, Carlsbad, CA, USA) was performed to remove trace endotoxin contaminant. Monoclonal mouse IgG against Stx1 (Stx1-13C4) and against Stx2 (Stx2-BB12) was purchased from Toxin Technology Inc. (Sarasota, FL, USA). The anti-Stx1 and anti-Stx2 antibodies recognize the B subunits of the respective toxins.

### 3.2. Cells

Raji cells were kindly provided by Prof. Andrea Bolognesi (Dipartimento di Medicina Specialistica, Diagnostica e Sperimentale, Sede di Patologia Generale, Università di Bologna, Bologna, Italy). Cells were cultured in RPMI 1640 medium (Lonza, Walkersville, MD, USA) containing antibiotics (60 U/mL penicillin, 60 μg/mL streptomycin, Cambrex, Walkersville, MD, USA) and supplemented with 4 mM l-glutamine (Sigma Aldrich, St. Louis, MO, USA) and 10% FBS (Lonza, Walkersville, MD, USA). Cultures were kept in an incubator at 37 °C in a water-saturated 5% CO_2_ atmosphere in air. Before seeding, cells were routinely counted after proper dilution, and cell death was monitored by calculating the percentage of cells permeated by 0.06% trypan blue.

### 3.3. Human Blood Samples

Sera were obtained from healthy donors or from children with bloody diarrhea caused by STEC. All subjects or their parents gave their informed consent for inclusion before they participated in the study. The study was conducted in accordance with the Declaration of Helsinki, and the protocol was approved by the Ethics Committee of the Fondazione IRCCS (Istituto di Ricovero e Cura a Carattere Scientifico) Ca’ Granda Ospedale Maggiore Policlinico, Milan, Italy (18 May 2010). The rapid enrollment assays for Stx detection in patients’ feces were an immunochromatographic test (Immunocard STAT EHEC, Meridian Bioscience Inc., Milan, Italy) or a PCR-based reverse dot blot. Detection of *stx1*, *stx2* and *eae* genes in enrichment cultures of feces was performed by real-time PCR (RT-PCR) as previously described [45].

### 3.4. Statistics

Data analysis was performed with GraphPad Prism 5. Continuous variables were described through means and SD. Differences in continuous variables were tested with the *t*-test after controlling the normality of their distribution. A value of *p* < 0.05 was considered statistically significant. Correlation between variables was assessed using the Pearson correlation coefficient.

## 4. Conclusions

Different rapid immunological methods (ELISA, immunochromatography, reverse passive latex agglutination test) for the detection of Stx in feces have been proposed for the rapid diagnosis of STEC infections. Conversely, the method developed in the present paper is aimed at detecting the functional activity of Stx in the serum of patients with a diagnosis of STEC infection. This will allow one to study the dynamics of the natural course of STEC-mediated diseases, by monitoring the main pathogenetic factors involved in the transition from hemorrhagic colitis to HUS. Although the sensitivity of the method for Stx2 is lower with respect to Stx1, the herein reported detection of the activity of Stx2 in patients’ sera represents a clear-cut demonstration that this HUS-associated toxin type is operative during STEC infections in humans, despite the presence of blood protecting factors, such as HuSAP. Different sound methods (direct or indirect flow cytometric assays, ELISA) [16,17,22,24], which measure the presence of Shiga toxins in different blood components (neutrophils, platelets, monocytes, erythrocytes, leukocyte-platelets aggregates, microvesicles) or in sera (cell-based methods) [28,29,30,41], have been described. However, the few based on the detection of the functional activity of the toxic molecule are time consuming (overnight incubation of cells) [28,29,30,41] and/or require modified cell lines [41]. The method described in this paper is rapid (4 h), sensitive (limit of detection: 2 pg/mL of Stx1a and 100 pg/mL of Stx2a), specific (inhibition of protein synthesis by Stx), adapted to human sera, validated with monoclonal antibodies to Stx and utilizes commercially-available stable cells (Raji cells). The main drawback is related to the use of radioactive precursors, which mandatorily must be operated and managed in radioisotope-designated laboratories.

Shiga toxins fleetingly appear in patients’ sera during the intestinal phase as assayed by ELISA [22], while they are not detectable during the renal phase of the infection using the cell-based method, which measures their functional activity [28,29,30]. To the best of our knowledge, the application of our cell-based assay allowed the first demonstration of the presence of active free Stx in the sera of three patients who had concomitant detectable Stx in feces and suffered from bloody diarrhea caused by STEC. In Italy, a network connecting 53 pediatric units has been established since 2010, through a cooperation between the Center for HUS Control, Prevention and Management in Milan and the local (Regione Lombardia) and national (Istituto Superiore di Sanità, Rome) health authorities aiming at studying STEC infections in children during the prodromal intestinal phase. We are applying the assay described in the present paper in the framework of the activity of this network for the daily detection of free Stx in blood. The study of the kinetics of Stx in the different blood fractions (serum, neutrophils, monocytes, platelets, leukocyte-platelet aggregates, microvesicles) and in particular the interplay between their functional activity in sera and the presence in different blood fractions is of paramount importance to foster our understanding of the pathogenesis of diarrhea-associated HUS, since these powerful bacterial toxins represent the main factor involved in the development of HUS in STEC-infected children.
